# Invasive Breast Carcinoma With Sebaceous Morphologic Pattern Showing Lymph Node Macrometastasis: A Case Report

**DOI:** 10.7759/cureus.37365

**Published:** 2023-04-10

**Authors:** Juan Carlos Alvarez Moreno, Jing He

**Affiliations:** 1 Pathology, University of Texas Medical Branch at Galveston, Galveston, USA

**Keywords:** metastasis, sebaceous pattern, sebaceous carcinoma, invasive ductal carcinoma, breast cancer

## Abstract

Invasive ductal carcinoma of no special type can present with various patterns. It is not possible to diagnose them through imaging alone. Microscopic examination is necessary to accurately identify and characterize them. The sebaceous pattern was historically considered a distinct subtype of breast carcinoma. However, the number of cases is relatively small and the prognosis has not been fully established. In this paper, we present a case of invasive ductal carcinoma with focal sebaceous features, which had a macrometastasis to the axillary lymph nodes showing the sebaceous morphology.

## Introduction

Invasive ductal carcinoma of no special type (IDC-NST) may present with the rare sebaceous pattern, which was first reported by Van Bogaert and Maldague in 1977 as lipid-producing tumors of the breast [1]. It was previously considered a metaplastic carcinoma [2]. According to the World Health Organization (WHO) fourth edition, sebaceous carcinoma (SC) was classified as a distinct type of breast carcinoma among the rare special types [3]. It resembles skin adnexal tumors with sebaceous differentiation, but there is no evidence of cutaneous derivation. Due to the limited clinical evidence, the current fifth edition of the WHO classifies it as a special morphologic subtype of invasive breast carcinoma of no special type regardless of the extent of the pattern. Because of its rare occurrence, its behavior and prognosis are not well understood yet [4]. In this report, we present a case of breast carcinoma with sebaceous differentiation that caused metastasis to the axillary lymph node.

## Case presentation

A 56-year-old woman underwent routine mammography which revealed a focal asymmetry in the left breast. Ultrasonography showed an 8 mm hypoechoic mass with shadowing at the 1 o’clock position of the left breast. The skin appeared unremarkable. A subsequent ultrasound-guided core needle biopsy of the breast lesion demonstrated invasive ductal carcinoma. Immunohistochemistry studies showed positive staining for estrogen receptor (ER) and progesterone receptor (PR) and negative staining for Her2. The patient underwent a segmental mastectomy with a sentinel lymph node biopsy.

**Figure 1:**
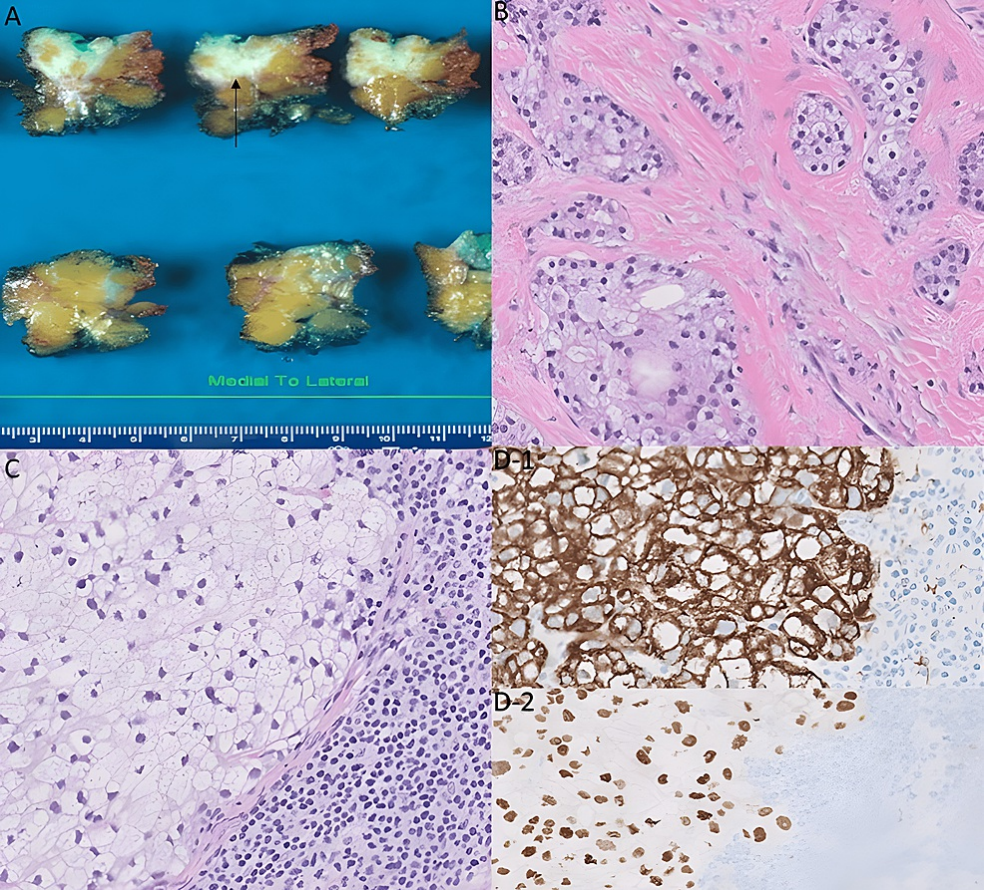
A: The gross examination showed a firm white mass lesion with infiltrative borders (arrow). B: The histologic examination of this mass lesion revealed solid growth of nests of tumor cells with abundant vacuolated cytoplasm and eccentrically located scalloped nuclei, which suggested sebaceous differentiation (magnification: 20×). C: Microscopic examination of the sebaceous pattern of breast carcinoma that metastasized to the axillary lymph node (magnification: 20×). D1-D2: Immunohistochemistry showing the tumor cells in the lymph node were positive for AE1/AE3 and expressed estrogen receptor (magnification: 20×).

During the macroscopic examination, a tan-white, firm, ill-defined mass measuring 15 mm was observed with no continuity with the overlying skin (Figure 1A). The histological examination revealed the presence of invasive ductal carcinoma and ductal carcinoma in situ (DCIS). The tumor was grade 2 using the modified Nottingham combined histologic grade. The tumor exhibited focal areas of solid growth and consisted of nests of tumor cells with vesicular and vacuolated cytoplasm. These findings were characteristic of sebaceous differentiation in invasive carcinoma and DCIS (Figure 1B). The invasive carcinoma cells exhibiting sebaceous differentiation comprised 10% of the tumor. The tumor cells neither reached the overlying skin dermis nor showed a pagetoid spread within the epidermis. Histological examination of three lymph nodes revealed one lymph node with a focus of metastatic carcinoma with sebaceous differentiation (Figure 1C). Immunohistochemistry studies performed on the lymph node showed AE1/AE3 positivity (Figure 1D-1), ER positivity (Figure 1D-2), and negative CD68 staining. The immunoprofile supported the conclusion that this metastasis originated from the primary tumor, which was diagnosed as IDC-NST with sebaceous differentiation. After surgery, the patient received radiation and adjuvant therapy. She is currently receiving treatment and will be followed up for six months after the completion of her therapy.

## Discussion

SC is a rare malignant tumor that originates from the sebaceous glands and can occur in any body site where sebaceous glands are present [4]. SC is most commonly found in the periocular area. Although extraocular SC is infrequent, it can occur in the major salivary glands, oral mucosa, breasts, lungs, and ovaries [4]. Primary breast SC is an extremely rare type of breast tumor and is classified as a sebaceous pattern of IDC-NST in the fifth edition of the WHO breast tumor classification system. This pattern is characterized as a breast carcinoma with sebaceous differentiation originating from the mammary gland tissue, with no evidence of derivation from cutaneous adnexa [5]. The origin of sebaceous cells in breast carcinoma is not fully understood [6]. There are hypotheses that attempt to explain sebaceous differentiation, including the existence of a ductal reserve cell capable of sebaceous differentiation, as well as the displacement of the embryonic group of epidermal cells into the breast parenchyma [6]. The morphology of the tumor is thought to result from the malignant transformation of these cells [7,8]. Our identification of sebaceous metaplasia in the DCIS of our case aligns with this hypothesis. However, additional molecular and genetic studies are necessary to fully support this theory.

Lynch syndrome (LS) is caused by a germline mutation in DNA mismatch repair genes (MMR), causing microsatellite instability [9]. This syndrome causes cancer, such as colorectal carcinoma and others. Using immunohistochemical stains, we can detect the mutations in MMR as a loss of nuclear expression in *MLH1*, *PMS2*, *MSH2*, and *MSH6 *in the tumor [9]. The Muir-Torre syndrome is a variant of the LS that causes extraocular SCs [10]. However, according to the literature, there was no loss of expression of MMR in invasive ductal carcinomas with sebaceous differentiation, indicating that these sebaceous features are not associated with Muir-Torre syndrome [7,11].

Histologically, the sebaceous pattern of breast carcinoma is characterized by lobulated or nested proliferation of tumor cells with abundant vacuolated cytoplasm, resembling mature sebocytes. The nuclei can vary from small, monomorphic, darkly staining small cells to pleomorphic large cells with eccentric nuclei, mostly, and prominent nucleoli [5]. Mitotic figures may be numerous, as reported in a case series by Svajdler et al. in which mitotic figures ranged between 5 and 39 per 10 high-power fields [12]. Our case showed similar features to the previously reported cases, except for the absence of pleomorphic large cells and the low mitotic rate.

The differential diagnosis includes lipid-rich carcinoma, glycogen-rich clear cell carcinoma, and skin SC [6]. The tumor must originate from breast gland parenchyma and lack evidence of primary cutaneous adnexal sebaceous glands [5]. In our case, there was no evidence of carcinoma involving the overlying skin. The lipid-rich carcinoma shows similar microscopic features to the sebaceous pattern. However, unlike the sebaceous pattern which shows a compact lobulated solid growth pattern and finely vacuolated cells, lipid-rich carcinomas infiltrate similarly to a regular invasive ductal carcinoma with a watery-clear cytoplasm and less conspicuous vacuolization [12]. In lipid-rich carcinoma, cytoplasm may stain positive with Sudan III or Oil red in the fresh material [6]. Most cases are negative for ER and PR, and Her2 positivity is variable in the literature [13]. Although we could not perform fat stains, the cytoplasm showed coarse vacuoles that are less subtle than lipid-rich subtypes [7]. The glycogen-rich clear cell pattern is characterized by abundant cytoplasmic glycogen which is positive for periodic acid-Schiff staining. The nuclei are round to oval, with clumped chromatin and prominent nucleoli. This pattern has shown to have 44.8% of cases negative for ER and Her2. Additionally, most cases are also negative for PR [14-16].

The sebaceous pattern of breast carcinoma shows a high expression rate of ER and PR and a low expression of Her2. Most of the sebaceous pattern of breast carcinoma shows a luminal phenotype and has a favorable prognosis with a low potential for metastatic spread [17]. Hormonal therapy can be a good treatment option. However, some cases demonstrated more aggressive features with lymph nodes and distant metastasis. Studies by Svadjler et al. [12], Hisaoka et al. [18], and Murakami et al. [19] have reported axillary lymph node metastasis. The metastatic sites such as skin, bone, liver, lung, and mediastinum have been reported [12]. In our case, the tumor cells demonstrated positivity for ER and PR and negativity for Her2, with the presence of lymph node metastasis, but no evidence of distant metastasis.

## Conclusions

We report a rare case of breast carcinoma with a sebaceous pattern with lymph node metastasis. It was surprising that despite such a small focus in the breast tissue, it manifested as a macrometastasis. This could suggest a more aggressive behavior similar to some cases described in the literature. The prognosis remains unknown due to the limited number of cases reported so far. Further studies are needed to better understand the prognosis of patients with sebaceous differentiation of breast carcinoma.
